# Positive Effects of Extra-Virgin Olive Oil Supplementation and DietBra on Inflammation and Glycemic Profiles in Adults With Type 2 Diabetes and Class II/III Obesity: A Randomized Clinical Trial

**DOI:** 10.3389/fendo.2022.841971

**Published:** 2022-05-02

**Authors:** Erika Aparecida Silveira, Lorena Pereira de Souza Rosa, Danilo Pires de Resende, Ana Paula dos Santos Rodrigues, Adeliane Castro da Costa, Andréa Toledo de Oliveira Rezende, Matias Noll, Cesar de Oliveira, Ana Paula Junqueira-Kipnis

**Affiliations:** ^1^ Graduate Program in Health Sciences, Faculty of Medicine, Federal University of Goiás, Goiás, Brazil; ^2^ Department of Epidemiology and Public Health, University College London, London, United Kingdom; ^3^ Federal Institute of Goiás, Campus Goiânia, Goiás, Brazil; ^4^ Department of Biosciences, Institute of Tropical Pathology and Public Health, Federal University of Goiás, Goiás, Brazil; ^5^ Instituto Federal Goiano, Campus Ceres, Goiás, Brazil

**Keywords:** obesity, fasting insulin, interleukin, inflammatory response, IL-1α, adiponectin, TNFα, diabetes

## Abstract

**Background:**

Evidence on the effects of dietary interventions on inflammatory markers in individuals with obesity and type 2 diabetes mellitus (T2DM) is scarce. Our study evaluated the effects of extra-virgin olive oil alone and in combination with a traditional Brazilian diet on inflammatory markers and glycemic profiles in adults with both T2DM and class II/III obesity.

**Methods:**

Adults aged 18-64 years with T2DM and class II/III obesity were randomized into two intervention groups: 1) extra-virgin olive oil only and 2) extra-virgin olive oil + a traditional Brazilian diet (OliveOil+DietBra). Data on sociodemographic characteristics, lifestyle, anthropometry, biochemical markers and inflammatory markers were collected. The primary outcomes were glycemic parameters and inflammatory markers. The body mass index (BMI) and weight were the secondary outcomes.

**Results:**

Forty individuals with T2DM and class II/III obesity were enrolled, and 34 (85%) completed the intervention course. The intake of olive oil was 37.88 ± 12.50 mL/day in the olive oil group and 37.71 ± 12.23 mL/day in the OliveOil+DietBra group, with no significant difference between groups (p = 0.484). Compared to the olive oil only group, the OliveOil+DietBra group had significantly lower levels of fasting insulin (p = 0.047) at the end of the intervention, whereas the other glycemic parameters were not altered. In the OliveOil+DietBra group, serum levels of inflammatory cytokines, IL-1α (p = 0.006) and adiponectin (p = 0.049) were lower and those of TNFα were higher (p = 0.037). There was a significant reduction in BMI and weight compared to the baseline values in the OliveOil+DietBra group (p = 0.015).

**Conclusions:**

The intervention with OliveOil+DietBra effectively decreased the levels of fasting insulin, IL-1α and adiponectin, suggesting its beneficial role in improving the inflammatory profiles and fasting insulin levels in adults with class II/III obesity and T2DM.

**Clinical Trial Registration:**

ClinicalTrials.gov, identifier: NCT02463435

## Introduction

Obesity is strongly associated with type 2 diabetes mellitus (T2DM) (1). Adipose tissue can secrete proinflammatory cytokines such as interleukin 6 (IL-6) and tumor necrosis factor alpha (TNFα), which contribute to insulin resistance (IR) (2). Adiponectin is also involved on inflammatory response, insulin sensibility and energetic balance. Chronic inflammation associated with obesity contributes to increased prevalence of T2DM (3, 4). Chronic inflammatory responses may also be induced by atherosclerosis, diabetes, rheumatoid arthritis, inflammatory bowel disease, cancer, or neurodegenerative disease (5). The Mediterranean diet (DietMed), which includes an adequate amount of extra-virgin olive oil, has been shown to improve the pathophysiology associated with obesity, T2DM, cardiovascular diseases (6–10), and reduce inflammatory cytokines (5, 11–13). The major cytokines involved in the inflammatory pathophysiology i.e. TNF-α and IL-6 are reduced in obese individuals receiving the DietMed intervention (14).

Previous studies of T2DM individuals with different ranges of body mass index (BMI) reported beneficial effects of DietMed on glycemic profiles (12, 15–18). The effects of olive oil on inflammatory markers in eutrophic, overweight and obese individuals with T2DM have also been reported (3, 19–24). These promising benefits of DietMed may be related to the anti-inflammatory and antioxidant properties of extra-virgin olive oil, as the latter is rich in monounsaturated fatty acids (MUFAs), polyphenols, hydrocarbons, phytosterols, and tocopherols (25). However, most of these studies were conducted in Mediterranean countries and the effect of an olive oil-rich diet may not be extrapolated to other dietary patterns. In addition, it may be difficult to implement DietMed in other countries where such a diet is not part of their usual diet. Furthermore, there is a lack of randomized clinical trials evaluating the role of olive oil alone or in combination with another dietary intervention pattern in reducing inflammatory markers among adults with T2DM and class II/III obesity.

Given the serious complications associated with T2DM and severe obesity and the limited interventions in this area, this study aimed to evaluate the effects of extra-virgin olive oil and the traditional Brazilian diet (DietBra) (26, 27) on inflammatory markers and glycemic profiles in adults with both T2DM and class II/III obesity. This study is clinically relevant since it can contribute to improve the treatment of individuals with both clinical conditions.

## Materials and Methods

### Study Design

This study is part of the DietBra Trial, which is a randomized clinical trial (RCT) to assess the effects of the traditional Brazilian diet (DietBra) with extra-virgin olive oil supplementation on several health outcomes in T2DM and class II/III obesity. The study was conducted at the Clinical Research Unit of the Federal University of Goiás (UFG), Goiânia, Goiás, Brazil (26–30). It was conducted according to the recommendations of the Consolidated Standards of Reporting Trials (CONSORT). This trial was permitted by the Ethics Committee of the Universidade Federal de Goiás (protocol no. 747.792/2014) and conducted in accordance with Declaration of Helsinki principles. All participants consented to participate.

### Participant Recruitment

Individuals with class II/III obesity (BMI ≥ 35 kg/m^2^), aged 18-64 years and living in the Goiania metropolitan area were referred to the Nutrition and Severe Obesity Outpatient Clinic at UFG *via* the primary care network of the Unified Health System. This is the only outpatient clinic specialized in class II/III obesity in this area. Individuals with T2DM were identified by their glycemic profiles (glycosylated hemoglobin ≥ 6.5% or fasting glucose ≥ 126 mg/dL) as well as by the use of prescribed hypoglycemic medications (1).

Individuals diagnosed with type 1 diabetes, lactating or pregnant, who had undergone bariatric surgery, with special needs, who had lost >8% of body weight in the last 3 months, receiving any nutritional or medical intervention for weight reduction, receiving any nutritional treatment in the recent two years, using insulin or anti-obesity drugs, or with food intolerance to some types of vegetable oil were excluded.

The sample size was determined based on previous RCT studies relate to individuals with chronic diseases and obesity that assessed similar outcomes and also on the central limit theory. The latter proposes that for studies with samples of 50 individuals or less, it is possible to find statistical differences where they exist (31).

### Randomization and Quality Control

**Figure 1:**
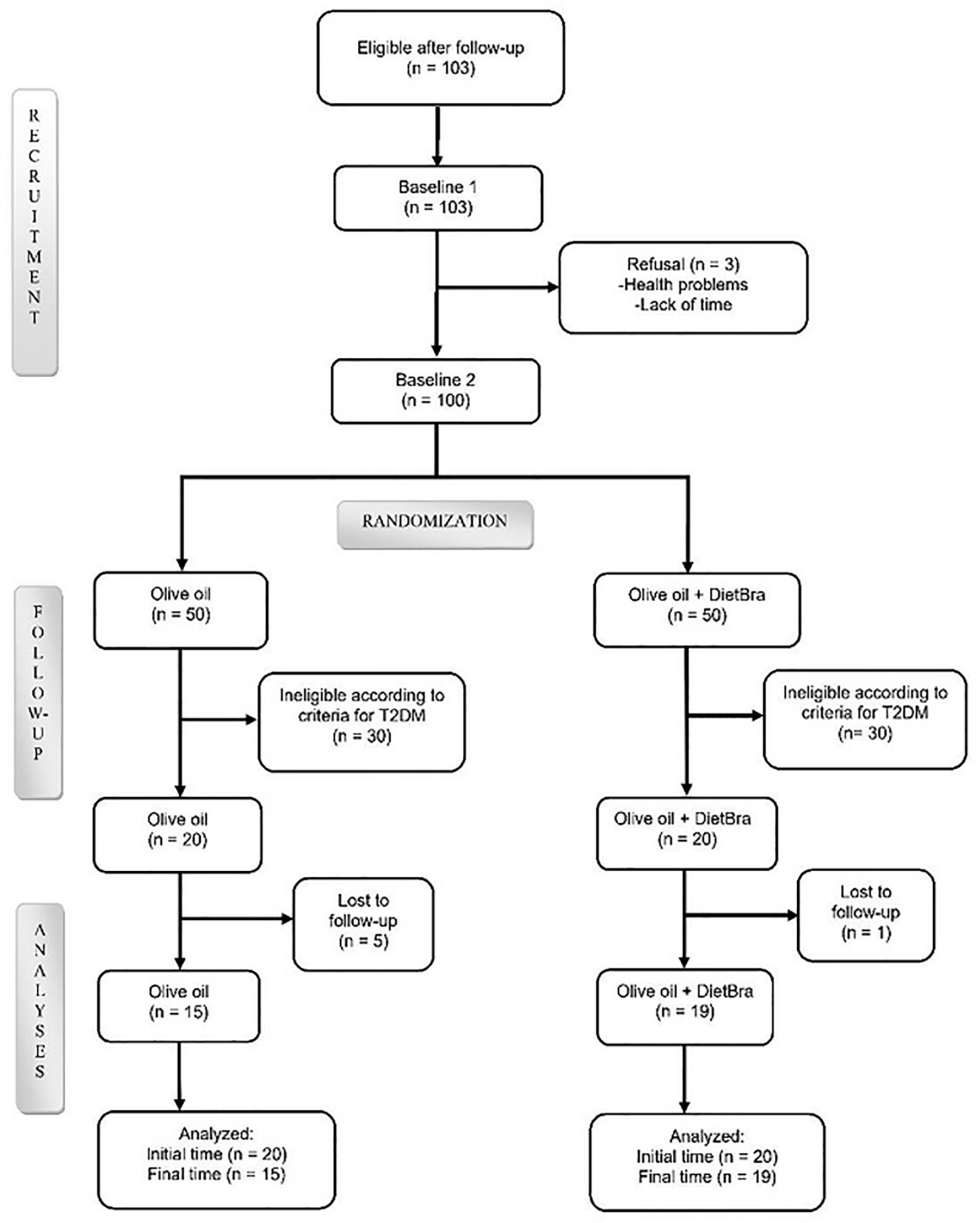
Flowchart of participant selection. Olive extra-virgin olive oil; DietBra, the traditional Brazilian diet.

A total of 103 participants were initially screened for T2DM. After excluding individuals who did not meet the eligibility criteria, 40 participants were randomized at baseline into two intervention groups, with a 1:1 allocation and parallel intervention, using a randomization list generated by the site www.randomization.com. The groups were: 1) extra-virgin olive oil and 2) extra-virgin olive oil + DietBra (**Figure 1**).

The random allocation sequence was performed by the senior researcher (EAS). LPRS enrolled the participants according to the criteria defined in the clinicaltrial.gov registration, and trained nutritionists assigned participants to interventions.

Although, clinical trials with the nutritional interventions are difficult to be completely blinded, we applied some procedures to avoid bias. The both groups had no contact with each other, and each group were scheduled to visit the clinic on different days. Throughout the data collection period, the name “extra-virgin olive oil” was substituted for “food supplement” to ensure that the participants were not aware of the type of intervention they were assigned to (32, 33). To ensure anonymity of the intervention, each extra-virgin sachet of olive oil was packaged and delivered with no technical information. Therefore, participants did not specifically know which food supplement they were receiving. The statistician was also blinded regarding the type of interventions during data analyses.

Data quality was guaranteed by training the researchers how to avoid loss to follow-up and standardization all protocols. All intervention groups returned every four weeks, with a total follow-up time of 12 weeks. Study variables were collected using a structured, pre-tested and standardized questionnaire. The questionnaires were counted by different researchers after each consultation. The adherence to the Brazilian diet was evaluated by nutritionists during follow-up consultations in which they applied a 24-hour recall to verify eating behaviour and adherence to the prescribed diet plan. The nutritionists were well trained in standardize interventions, diet plan prescriptions, and to check if any patient had difficulty in adherence. All these aspects associated with dietary intake and adherence to the Brazilian diet were reinforced and discussed during the consultation to motivate patients to follow the diet plan.

### Dietary Interventions: DietBra and Extra-Virgin Olive Oil

DietBra is a healthy dietary pattern defined as consuming unprocessed foods in at least four meals per day (34). Traditional Brazilian foods including rice, beans, lean meat, and vegetables, either raw or cooked, are recommended for the main meals (lunch and dinner). Consumption of seasonal fruits during the day is also recommended, while breakfast and/or afternoon snack may consist of milk, dairy products, or coffee (26, 27). DietBra contains adequate amounts of vitamins, minerals, fiber, and low amounts of *trans* and hydrogenated fats, all of which characterize a healthy dietary pattern. DietBra was based on the common Brazilian healthy food culture before the nutritional transition process (35). Moreover, it is accessible and economical for the general public in Brazil and many other countries (36). More information on the comparison between the Mediterranean and the Brazilian diets can be found in a previous study of the DieTBra trial (26).

The total energy value (TEV) of DietBra was estimated using a specific formula based on the resting energy expenditure (REE) and the fat-free mass for severely obese patients (37). The total energy expenditure (TEE) was calculated by multiplying the REE by the activity factor (AF) plus the thermic effect of food (TEF) (38). The AF of each participant corresponds to the physical activity level according to the Global Physical Activity Questionnaire (39).

The TEV of DietBra was calculate based on the goal of a 5-10% reduction of the initial weight according to the BMI range: for 35-40 kg/m^2^, 5-6% reduction; for 40-50 kg/m^2^, 7-8% reduction; and for >50 kg/m^2^, 9-10% reduction. The Dietary Reference Intakes developed by the Institute of Medicine (40) were used for macronutrient distributions of the diet plans. The DieTBra groups received an individualized and prescribed diet plan to follow and had consultations with a dietitian/nutritionist while the olive oil + DietBra did not receive this intervention.

The olive oil + DietBra group received DietBra and a daily supplementation of 52 mL extra-virgin olive oil (468 kcal/day) (28, 29, 41, 42). Each laminated sachet contained 13 mL of cold-pressed commercial extra-virgin olive oil with <2% acidity, which facilitates its consumption as well as avoiding portioning errors. Four sachets were consumed per day, with a suggestion of two sachets at lunch and two at dinner. Although the caloric value of the olive oil was reduced in the amounts of prescribed foods, this group still preserved a high-fat dietary prescription (around 45%).

Participants from olive oil group were advised to keep their usual diet and consumed the same amount (52 mL/day) of extra-virgin olive oil to allow the assessment of the effect of olive oil supplementation alone on the primary and secondary outcomes. Full and empty sachets were collected and counted at each monthly return visit to measure compliance and calculate the amount of consumed extra-virgin olive oil. For both groups, the sachets were delivered at the end of each visit in enough quantities for the monthly consumption. The intervention lasted longer than 12 weeks.

The adherence to olive oil consumption was assessed by counting the returned sachets of olive oil by participants, empty or not. The adherence to the DietBra was evaluated during the consultation with trained nutritionists. At baseline we performed three 24-hour recalls to estimate the dietary intake before the intervention. The main objective of this approach was to estimate changes in macronutrients and calorie intake during 12 weeks of follow-up.

### Sociodemographic Characteristics, Lifestyle and Anthropometry

Sociodemographic variables include sex, age, education (years of schooling), and economic classification defined by the Brazilian Association of Population Studies (43). Smoking status was classified according to the Pan American Health Organization: former smoker, current smoker, and non-smoker (never smoked).

Anthropometric measurements were collected following a standard protocol. For weight measurements, the Welmy digital scale (capacity, 200 kg; accuracy, 100 g) was used. Height was measured using a stadiometer (accuracy, 0.1 cm). Weight and height were used to calculate BMI (44).

### Biochemical Examinations and Inflammatory Markers

The diagnosis of T2DM was determined by the use of oral hypoglycemic agents and/or fasting glucose (≥126 mg/dL) and/or glycosylated hemoglobin (≥6.5%) (1). The following markers were also analyzed: fasting insulin, homeostasis model assessment of insulin resistance index (HOMA-IR index), and C-reactive protein. Blood was collected after 12 hours of fasting. HOMA-IR result was defined by: HOMA-IR = fasting serum insulin (U/mL) × fasting plasma glucose (mmol/L)/22.5.

Interleukins (IL)-4), IL-10, monocyte chemoattractant protein 1 (MCP-1), and TNFα were examined using the Bio-Plex^®^ Pro Human Cytokine 27-Plex kit (Bio-Rad, Hercules, CA) per manufacturer’s instructions in the Bio-Plex 200 System provided by the Oswaldo Cruz Foundation, Rio de Janeiro. Enzyme-linked immunosorbent assays were used to measure adiponectin (Human Adiponectin PicoKine™ ELISA Kit, Boster Bio, Pleasanton, CA), leptin (Human Leptin PicoKine™ ELISA Kit, Boster Bio, Pleasanton, CA), IL-1β, and IL-6 (Human IL-1β PicoKine™ ELISA Kit and Human IL-6 PicoKine™ ELISA Kit, Boster Bio, Pleasanton, CA, respectively) per manufacturer’s instructions. All measurements were performed at the Laboratory of Immunopathology of Infectious Diseases.

### Statistical Analyses

The database was constructed with double entries using EpiData^®^ (v3.1) for checking consistency and information validation. Data were analyzed by descriptive and inferential tests. Descriptive analysis include absolute and relative frequencies, means and standard deviations. The outcomes (glycemic parameters, inflammatory markers, BMI and weight) were first analyzed using the Shapiro-Wilk test to assess normality. Outcomes between the baseline and the end of the intervention were assessed by the Mann-Whitney test (for nonparametric data) and independent *t*-test (for parametric data) for each group. Outliers were identified using the ROUT method in GraphPad Prism 7, which were replaced by the mean and median values were computed (p < 0.05). All analysis were performed by Stata/SE 16.0 software (Stata Corp, USA).

## Results

A total of 40 participants with both T2DM and class II/III obesity were eligible to entry the study. After randomization, we found no sociodemographic characteristics or study outcomes differences between the groups at baseline (**Table 1**).

**Table 1:** Baseline sample characteristics, the DietBra Trial.

Variables	Total n (%)	Olive oil (n = 20) n (%)	Olive oil + DietBra (n = 20) n (%)	*p* value
Gender				
Female	34 (85)	18 (90)	16 (80)	0.376
Male	6 (15)	2 (10)	4 (20)	
Age (years)				
20-40	18 (45)	11 (55)	7 (35)	0.204
41-60	22 (55)	9 (45)	13 (65)	
Education (years)				
≤8	22 (55)	9 (45)	13 (65)	0.204
≥9	18 (45)	11 (55)	7 (35)	
Economic class				
A-B	5 (12,5)	3 (15)	2 (10)	0.856
C	26 (65)	13 (65)	13 (65)	
D-E	9 (22,5)	4 (20)	5 (25)	
Smoking				
Never smoked	25 (62,5)	13 (65)	12 (60)	0.744
Smoker/ex-smoker	15 (37,5)	7 (35)	8 (40)	
	**Mean ± SD**	**Mean ± SD**	**Mean ± SD**	***p* value**
Weight (kg)	117.63 ± 20.45	114.64 ± 19.16	120.63 ± 21.74	0.092
BMI (kg/m^2^)	46.5 ± 7.3	45.9 ± 7.42	47.1 ± 7.3	0.633
% Fat	52.21 ± 4.10	52.6 ± 3.10	51.8 ± 5.01	0.475
Fasting glucose (mmol/L)	125.1 ± 35.1	126.6 ± 34.4	123.5 ± 36.5	0.115
Fasting insulin (U/mL)	22.3 ± 11.2	24.1 ± 11.7	20.5 ± 10.7	0.158
HOMA-IR	7.6 ± 4.6	7.71 ± 4.43	7.5 ± 4.8	0.406
HbA1c (%)	7.5 ± 1.62	7.08 ± 1.44	7.84 ± 1.73	0.142
CRP (mg/dL)	6.60 ± 6.20	6.30 ± 6.59	6.90 ± 5.92	0.398
TNFα (pg/mL)	2.84 ± 4.83	0.69 ± 3.07	8.97 ± 21.89	0.102
IL-1α (pg/mL)	245.6 ± 95.5	271.2 ± 101.4	220.1 ± 84.1	0.067
IL-6 (pg/mL)	4.75 ± 6.60	4.53 ± 6.58	4.97 ± 6.79	0.615
MCP-1 (pg/mL)	0.16 ± 0.39	0.18 ± 0.43	0.13 ± 0.36	0.464
Adiponectin (ng/mL)	7.53 ± 7.05	8.02 ± 7.22	6.86 ± 7.04	0.231
Leptin (pg/mL)	4156.3 ± 188.8	4151.5 ± 191.2	4161.1 ± 191.3	0.989
IL-10 (pg/mL)	10.8 ± 7.7	9.09 ± 7.03	12.5 ± 8.11	0.426

DietBra, the traditional Brazilian diet; SD, standard deviation; BMI, body mass index; HOMA-IR, homeostatic model assessment of insulin resistance; HbA1c, glycated hemoglobin; CRP- C, reactive protein; TNFα, tumor necrosis factor alpha; IL-1α, interleukin 1 alpha; IL-6, interleukin 6; MCP-1, monocyte chemoattractant protein 1; IL-10, interleukin 10.

At baseline the protein intake (% TEV) in the Oil Olive group was 18.1 ± 4.3 (Mean ± SD), in the Olive Oil + DieTBra group was 18.7 ± 4.7 (Mean ± SD) and the energy intake (kcal/day) of the Oil Olive group was 1674.8 ± 610.8 (Mean ± SD), in the Olive Oil + DieTBra group was 1701.1 ± 1021.1 (Mean ± SD). There was no statistical difference in both groups.

During the intervention, the intake of olive oil was 37.88 ± 12.50 mL/day for the olive oil group and 37.71 ± 12.23 mL/day for the olive oil + DietBra group, with no significant difference between groups (p = 0.484).

**Figure 2:**
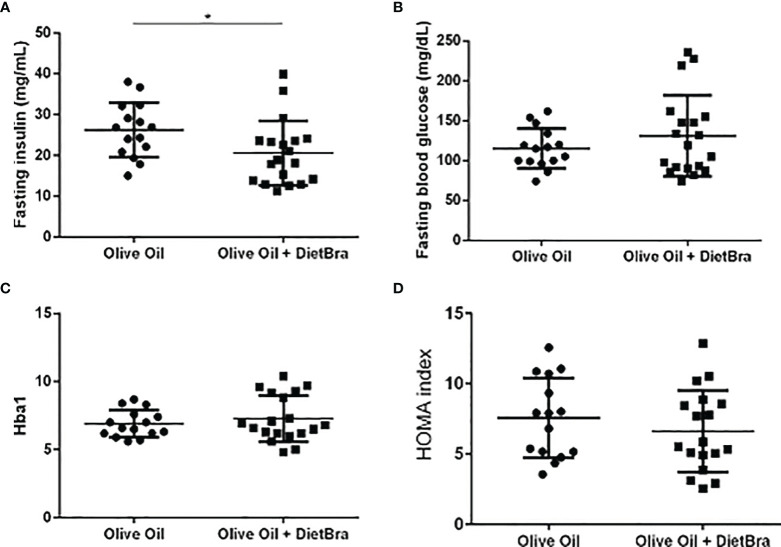
Comparisons of glycemic parameters at the end of the intervention between two intervention groups. **(A)** Fasting insulin; **(B)** Fasting blood glucose; **(C)** HbA1C (glycosylated hemoglobin); **(D)** Homeostasis Model Assessment of Insulin Resistance Index (HOMA-IR index). Independent *t*-test used to compare all variables between groups. *p=0.019.

**Figure 3:**
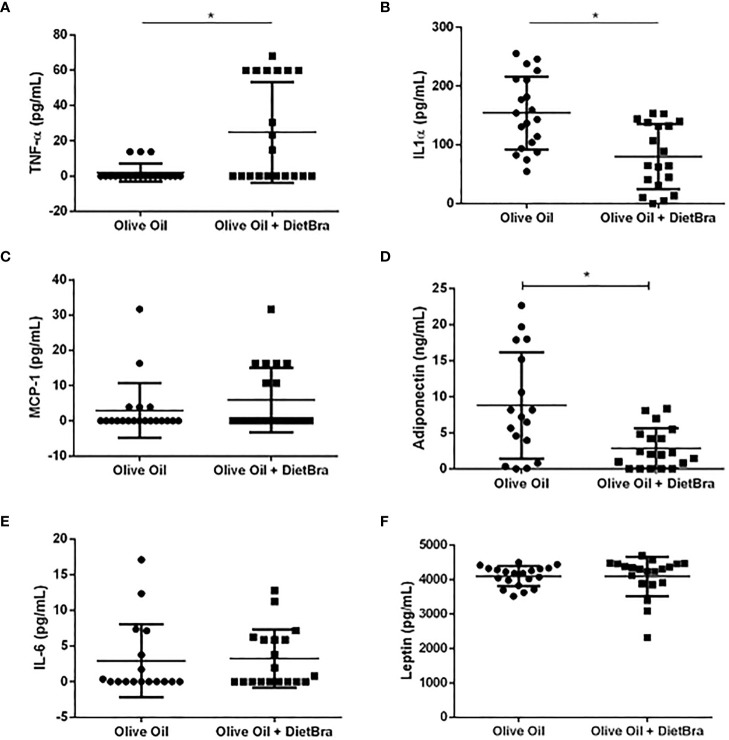
Comparisons of inflammatory biomarkers at the end of the intervention between two intervention groups. **(A)** TNF-α; **(B)** IL-1α; **(C)** MCP-1; **(D)** adiponectin; **(E)** IL-6; **(F)** leptin. Differences in TNF-α, MCP-1 adiponectin IL-6 and leptin were analyzed by the Mann-Whitney test and the difference in IL-lα was analyzed by the independent t-test. *p<0.05.

At the end of the intervention, the olive oil + DietBra group had significantly lower fasting insulin (p = 0.047) while other glycemic parameters did not differ compared to those in the olive oil only group (**Figure 2**). The serum levels of inflammatory cytokines IL-1α (p = 0.006) and adiponectin (p=0.0087) were lower and TNFα levels were higher (p = 0.037) in the olive oil + DietBra group compared to those in the olive oil only group (**Figure 3**).

**Figure 4:**
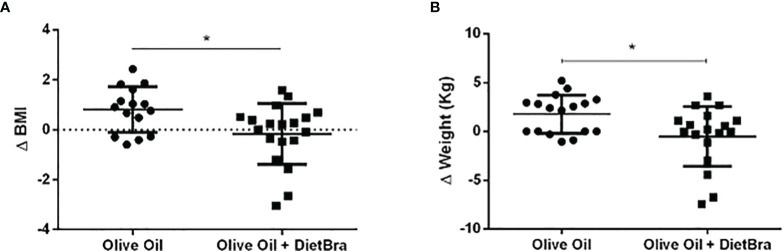
Comparisons of changes in anthropometric variables between intervention groups. **(A)** Changes in BMI analyzed by the independent t-test; **(B)** Changes in weight analyzed by the Mann-Whitney test Values below 0 indicate weight loss. *p<0.05.

Compared to the baseline values, significant reductions in BMI and weight were observed in the olive oil + DietBra group (p = 0.015) at the end of the intervention but not in the olive oil group (**Figure 4**). Numerical and descriptive data are presented in the **Tables 2**–**4**.

**Table 2:** Comparison between baseline and final follow-up variables in two intervention groups with class II/III obesity, Brazil, 2016 (n = 40).

Outcomes	Olive oil			Olive oil + DietBra					
	Mean ± SD		p		Mean ± SD				p	
	Before (n = 20)	After (n = 15)			Before (n = 20)		After (n = 19)			
Weight (kg)	114.64 ± 19.16	115.52 ± 20.17	0.061^#^		120.63 ± 21.74		118.96 ± 22.20		0.112^#^	
BMI (kg/m^2^)	45.9 ± 7.42	47.2 ± 7.87	0.613*		47.1 ± 7.3		46.81 ± 7.59		0.565*	
% Fat	52.6 ± 3.10	52.78 ± .339	0.294^#^		51.8 ± 5.01		51.41 ± 5.05		0.831^#^	
**Glycemic parameters**									
Fasting glucose (mmol/L)	126.6 ± 34.4	115.2 ± 25.1	0.233^#^		123.5 ± 36.5		127.6 ± 50.6		0.948^#^	
Fasting insulin (U/mL)	24.1 ± 11.7	26.23 ± 6.72	0.427^#^		20.5 ± 10.7		20.55 ± 7.88		0.629*	
HOMA-IR	7.71 ± 4.43	7.56 ± 2.83	0.977^#^		7.5 ± 4.8		6.51 ± 2.86		0.557^#^	
HbA1c (%)	7.08 ± 1.44	6.89 ± 0.99	0.683^#^		7.84 ± 1.73		7.27 ± 1.69		0.131^#^	
**Inflammation marker synthesized by hepatocytes**									
CRP (mg/dL)	6.30 ± 6.59	7.20 ± 7.92	1.000^#^		6.90 ± 5.92		6.94 ± 6.08		0.763^#^	
**Proinflammatory cytokines**									
TNFα (pg/mL)	0.69 ± 3.07	2.07 ± 5.03	0.317^#^		8.97 ± 21.89		24.8 ± 28.5		**0.041^#^**	
IL-1α (pg/mL)	271.2 ± 101.4	154.1 ± 62.5	0.001*		220.1 ± 84.1		89.1 ± 68.6		0.001*	
IL-6 (pg/mL)	4.53 ± 6.58	2.93 ± 5.12	0.311^#^		4.97 ± 6.79		3.32 ± 5.38		0.315^#^	
**Chemokine**									
MCP-1 (pg/mL)	0.18 ± 0.43	1.24 ± 2.36	0.061^#^		0.13 ± 0.36		2.39 ± 3.33		**0.013^#^**	
**Adipokines**									
Adiponectin (ng/mL)	8.02 ± 7.22	6.85 ± 5.74	0.093^#^		6.86 ± 7.04		4.19 ± 5.28		**0.049** ^#^	
Leptin (pg/mL)	4151.5 ± 191.2	4094.6 ± 290.2	0.601^#^		4161.1 ± 191.3		4244.9 ± 297.4		0.156^#^	
**Anti-inflammatory cytokine**									
IL-10 (pg/mL)	9.09 ± 7.03	16.34 ± 13.68	**0.042^#^**		12.5 ± 8.11		18.24 ± 15.5		0.349*	

Olive oil, extra-virgin olive oil; olive oil + DietBra, extra-virgin olive oil + traditional Brazilian diet; SD, standard deviation; BMI, body mass index; HOMA-IR, homeostatic model assessment of insulin resistance; HbA1c, glycated hemoglobin; CRP, C-reactive protein; TNFα, tumor necrosis factor alpha; IL1α, interleukin 1 alpha; IL6, interleukin 6; MCP1, monocyte chemoattractant protein 1; IL10, interleukin 10. Significant value in bold (p < 0.05). *Paired Student’s t-test. ^#^Wilcoxon test (paired, nonparametric).

**Table 3:** Comparison of outcome variables at the end of intervention between two intervention groups with class II/III obesity, Brazil, 2016 (n = 34).

Outcomes	Final evaluation (mean ± SD)		
	Olive oil (n = 15)	Olive oil + DietBra (n = 19)	*p* value
Weight (kg)	115.52 ± 20.17	118.96 ± 22.20	0.808^#^
BMI (kg/m^2^)	47.2 ± 7.87	46.81 ± 7.59	0.989*
% Fat	52.78 ± .339	51.41 ± 5.05	0.710^#^
**Glycemic parameters**			
Fasting glucose (mmol/L)	115.2 ± 25.1	127.6 ± 50.6	0.989^#^
Fasting insulin (U/mL)	26.23 ± 6.72	20.55 ± 7.88	**0.019^#^**
HOMA-IR	7.56 ± 2.83	6.51 ± 2.86	0.270^#^
HbA1c (%)	6.89 ± 0.99	7.27 ± 1.69	0.615**^#^**
**Inflammation marker synthesized by hepatocytes**			
CRP (mg/dL)	7.20 ± 7.92	6.94 ± 6.08	0.769^#^
**Proinflammatory cytokines**			
TNFα (pg/mL)	2.07 ± 5.03	8.12 ± 9.24	**0.037^#^**
IL-1α (pg/mL)	154.1 ± 62.5	89.1 ± 68.6	0.006*
IL-6 (pg/mL)	2.93 ± 5.12	3.32 ± 5.38	0.741^#^
**Chemokine**			
MCP-1 (pg/mL)	1.24 ± 2.36	2.39 ± 3.33	0.305^#^
**Adipokines**			
Adiponectin (ng/mL)	6.85 ± 5.74	4.19 ± 5.28	**0.048** ^#^
Leptin (pg/mL)	4094.6 ± 290.2	4244.9 ± 297.4	0.070^#^
**Anti-inflammatory cytokine**			
IL-10 (pg/mL)	16.34 ± 13.68	18.24 ± 15.5	0.804^#^

SD, standard deviation; olive oil, extra-virgin olive oil; olive oil + DietBra, extra-virgin olive oil + traditional Brazilian diet; BMI, body mass index; HOMA-IR, homeostatic model assessment of insulin resistance; HbA1c, glycated hemoglobin; CRP, C-reactive protein; TNFα, tumor necrosis factor alpha; IL1α, interleukin 1 alpha; IL6, interleukin 6; MCP1, monocyte chemoattractant protein 1; IL10, interleukin 10.

Significant value in bold (p < 0.05). *Independent t-test. ^#^Mann-Whitney test (independent, nonparametric).

**Table 4:** Comparison of changes (delta) in outcome variables between two intervention groups with class II/III obesity, Brazil (n = 34).

Outcomes (final - initial)	Olive oil (n = 15)	Olive oil + DietBra (n = 19)	*p* value
	Mean ± SD	Mean ± SD	
Δ Weight (kg)	1.42 ± 4.39	-1.60 ± 3.33	**0.004** ^#^
Δ BMI (kg/m^2^)	0.81 ± 0.91	-0.16 ± 1.22	0.015*
Δ % fat	-0.28 ± 1.26	-0.11 ± 1.31	0.544^#^
**Glycemic parameters**			
Δ Fasting glucose (mmol/L)	-7.20 ± 26.85	-4.74 ± 36.4	0.510^#^
Δ Fasting insulin (U/mL)	2.18 ± 10.58	0.51 ± 10.22	0.644*
Δ HOMA-IR	0.02 ± 3.89	-1.24 ± 4.32	0.386*
Δ HbA1c (%)	-0.13 ± 1.05	-0.63 ± 1.58	0.302*
**Inflammation marker synthesized by hepatocytes**			
Δ CRP (mg/dL)	0.00 ± 5.07	-0.32 ± 4.67	0.787^#^
**Proinflammatory cytokines**			
Δ TNFα (pg/mL)	1.81 ± 5.24	7.38 ± 9.07	0.059^#^
Δ IL1α (pg/mL)	-117.12 ± 117.70	-135.53 ± 120.9	0.449^#^
Δ IL6 (pg/mL)	-1.16 ± 7.60	-1.90 ± 7.70	0.987^#^
**Chemokine**			
Δ MCP1 (pg/mL)	1.08 ± 2.32	2.27 ± 3.31	0.229^#^
**Adipokines**			
Δ Adiponectin (ng/mL)	-1.73 ± 8.54	-1.58 ± 7.85	0.684^#^
Δ Leptin (pg/mL)	-56.8 ± 285.1	-83.59 ± 333.18	0.160*
**Anti-inflammatory cytokine**			
Δ IL10 (pg/mL)	7.24 ± 14.98	5.76 ± 18.98	0.785*

Olive oil, extra-virgin olive oil; olive oil + DietBra, extra-virgin olive oil + traditional Brazilian diet; SD, standard deviation; BMI, body mass index; HOMA-IR, homeostatic model assessment of insulin resistance; HbA1c, glycated hemoglobin; CRP, C-reactive protein; TNFα, tumor necrosis factor alpha; IL1α, interleukin 1 alpha; IL6, interleukin 6; MCP1, monocyte chemoattractant protein 1; IL10, interleukin 10.

*Independent t-test.

^#^Mann-Whitney test (independent, nonparametric).

Bold values was to identify the associated variables (when the p-value was statistically significant).

**Table 2** shows no significant difference in IL-10 levels in both intervention groups. However, in **Table 1** we can observe that this anti-inflammatory cytokine had increased after the olive oil intervention. There was an increase in both intervention groups, but this increase was statistically significant only in olive oil group.

## Discussion

As far as we know, this is the first RCT to assess how extra-virgin olive oil and DietBra affects inflammatory parameters and glycemic profiles in adults with both T2DM and class II/III obesity. Our main findings showed that the supplementation with extra virgin olive oil combined with the DietBra can decrease fasting insulin, inflammation markers such as IL-1α and adiponectin, as well as weight and BMI in class II/III obese adults with T2DM. These results are clinically relevant and can contribute to the current knowledge on T2DM and severe obesity, as these two nutritional interventions are relatively inexpensive with easy adherence in many countries that have similar dietary patterns.

Previous studies of olive oil have been conducted in overweight/obese T2DM individuals. However, in these studies olive oil was delivered in capsules and compared to other sources of isolated fats such as fish oil (3, 23, 24). In our study, olive oil was offered as a real food, which is an important aspect in nutritional interventions. Real food may be more favorable and effective to be incorporated into one’s daily food routine compared to adding more olive oil capsules to T2DM individuals’ existing medications.

The reduction in BMI in the olive oil + DietBra group is corroborated by a systematic review and meta-analysis investigating DietMed, also rich in olive oil, that reported a significant reduction in weight and BMI (45). Thus, appropriate eating plans compatible with regional food habits should be emphasized, since there is a greater probability of diet adherence and related benefits (35, 46). Furthermore, a study of dietary patterns provides more accurate and relevant results than a study of nutrients in isolation (47, 48). The food intake, macronutrients, and caloric intake of the participants at baseline have been previous analyzed, which showed no significant differences between groups (26, 49).

Although the intervention group i.e. olive oil + DietBra had greater weight loss and BMI reduction, the percentage fat was not significantly reduced. Therefore, it is likely that this intervention impacted on lean mass reduction. Reduction on lean mass should be avoid not only in individuals with class II/III obesity but also everybody in a weight loss treatment or dietary patterns modification. Weight loss with no percentage of body fat loss can indicated lean mass reduction which could lead to sarcopenic obesity, an inadequate clinical outcome in adults such as the population study. The ideal weight loss occurs when there is more percentage fat reduction and lean mass preservation and it should be achieved through a physical activity routine including both resistance training and aerobic exercises. A high body fat percentage is also associated with adiponectin production and tissue insulin resistance which may lead to catabolic effects on muscle mass (50–53). Because the high-fat diet has been prescribed with the inclusion of a healthy oil (extra virgin olive oil) the hypothesis was that it could reduce the pro-inflammatory cytokines when consumed with a healthy diet such as the Traditional Brazilian Diet.

Despite the lack of significant difference in the reduction of IL-1α between groups, the observed reduction is clinically relevant in both interventions i.e. approximately 50% reduction compared to the baseline values. IL-1α is an important inflammatory mediator and regulates micro-inflammation observed in participants with obesity and/or diabetes.

Furthermore, this study did not find significant changes in IL-6 levels with the dietary interventions. A new meta-analysis review with thirty RCTs using olive oil presented a reduction in IL-6 levels compared to the controls (54). The main difference between this study and others is the clinical characteristics of the participants. On this study the participants have both severe obesity and T2DM, which might be associated with different blood cytokine profiles that may hinder substantial reductions in cytokines levels in response to the dietary interventions. Therefore, our findings demonstrate that the understanding of inflammation in chronic diseases, such as diabetes and obesity, is rather limited and the anti-inflammatory effect of olive oil outside the context of DietMed requires further investigation (18, 55).

TNFα is an important inflammatory mediator normally present in high concentrations in the bloodstream of obese individuals (56). However, in this study, TNFα did not decrease at the end of the intervention in the olive oil + DietBra group. Notably, a positive correlation between TNFα levels and the development of insulin resistance has been previous reported in obese individuals (57). It is difficult to explain why the olive oil + DietBra intervention reduced the serum levels of insulin but not TNFα. A possible explanation may be the fact that TNFα is produced mainly by the adipose tissue in individuals with obesity (58), and there was no significant change in the percentage of body fat in this group despite the reduction in BMI. As a result, the intervention may not be able to affect the production of TNFα in these individuals. Additionally, the onset of insulin resistance is a complex process that involves various metabolic pathways (59). The improved serum levels of insulin by olive oil + DietBra may be mediated by factors other than TNFα, since other inflammatory markers were also reduced.

In this study, the olive oil + DietBra group had increased levels of TNF-α and decreased levels of adiponectin, which is consistent with previous research indicating that TNF-α suppresses adiponectin dimerization and secretion in adipocytes (60). Moreover, mice deficient in adiponectin presented higher levels of TNF-α in the blood, which was reduced when treated with adiponectin (61). Besides that, we highlight that there are controversies regarding the effect of olive oil on adiponectin and TNF-α levels. A study has shown that oleic acid in combination with the antioxidant phenol hydroxytyrosol prevented the suppression of adiponectin induced by TNF-α in adipocytes (62). However, whether this combination could be reflected in a diet remains debatable. On the other hand, Luo et al. (63) showed that treating adipocytes with oleic acid reduced the mRNA levels of adiponectin. Therefore, the role of olive oil rich in oleic acid in increasing TNF-α and reducing adiponectin needs further investigation.

Unfortunately, the analysis of covariance evaluating the influence of covariates (e.g. BMI and glycemic profiles) on the outcomes in both intervention groups could not be performed considering that the following requirements were not met: 1) the study outcomes were not normally distributed, and 2) no linear relationship existed between the covariates and the response variable (64).

The absence of a control group with no dietary intervention (e.g. Brazilian diet group alone) in this study could be a potential limitation. However, having a “no treatment” group is not ethically feasible for this population. For some inflammatory markers such as MCP-1, the methodology used may have not been efficient for the evaluation in this specific group, since the values from several individuals were below the detection limit. The technique used for the measurement of TNFα was the same used for the measurement of IL-10, monocyte chemoattractant protein 1 (MCP-1), through the Bio-Plex ^®^ Pro Human Cytokine 27-Plex kit (Bio-Rad, Hercules, CA), according to the manufacturer’s instructions Bio-Plex 200. It is a technique used worldwide and is therefore widely recommended. There is a limit of detection of the test, which for TNF-α is 1.13 pg/ml, according to what was done in the BioRad (https://www.biorad.com/webroot/web/pdf/lsr/literature/Bulletin_6335.pdf). It is possible that the levels found in this group of obese people are below the detection levels of the Bio-Plex 200 curve, causing the values to be very low, according to the sensitivity of the test. Nevertheless, the study has several strengths. The nutritional intervention with DietBra was based on natural and minimally processed foods, and extra-virgin olive oil was supplemented in the food form, not in capsules. There was a low loss to follow-up rate (15.0%), considering that obese individuals with or without T2DM generally have low adherence to treatments (65, 66). Given the prevalence of around 4% of the general population, it is hard to perform studies with a large number of individuals with both class II/III obesity and T2DM.

## Conclusion

Olive oil combined with DietBra decreased fasting insulin, IL-1α and adiponectin, while inducing a reduction in BMI and weight in individuals with T2DM and class II/III obesity. Future studies should consider the use of different amounts of olive oil in combination with DietBra or another healthy dietary pattern, an intervention period longer than 12 weeks and, finally, assessing more inflammatory parameters.

## Data Availability Statement

The original contributions presented in the study are included in the article/supplementary material. Further inquiries can be directed to the corresponding author.

## Ethics Statement

The studies involving human participants were reviewed and approved by Ethics Committee of the Universidade Federal de Goiás (protocol no. 747.792/2014). The patients/participants provided their written informed consent to participate in this study.

## Author Contributions

Conceptualization, ES. Methodology, ES and LR. Formal analysis, ES, LR, DR, and MN. Investigation, ES, LR, APR, DR, and AC. Resources, ES, APR, MN, and AJ-K. Data curation, ES, MN, and AJ-K. Writing, ES, LR, AC, MN, AJ-K, ATR, and CO. Review and editing, ES, MN, AJ-K, ATR, and CO. Supervision, ES and AJ-K. Project administration, ES. Funding acquisition, ES, APR, MN, and AJ-K. The principal investigator of the DietBra Trial is ES. All authors contributed to the article and approved the submitted version.

## Funding

FAPEG foundation provided partial funding for the DietBra Trial (grant number 201310267000003). CAPES foundation provided a doctoral scholarship. Instituto Federal Goiano provided partial funding. CO was supported by the Economic and Social Research Council (ESRC) (grant ES/T008822/1).

## Conflict of Interest

The authors declare that the research was conducted in the absence of any commercial or financial relationships that could be construed as a potential conflict of interest.

## Publisher’s Note

All claims expressed in this article are solely those of the authors and do not necessarily represent those of their affiliated organizations, or those of the publisher, the editors and the reviewers. Any product that may be evaluated in this article, or claim that may be made by its manufacturer, is not guaranteed or endorsed by the publisher.
